# Acceptance by Honey Bees of Wax Decontaminated through an Extraction Process with Methanol

**DOI:** 10.3390/insects14070593

**Published:** 2023-06-30

**Authors:** José Manuel Flores, Alba Luna, Antonio Rodríguez Fernández-Alba, María Dolores Hernando

**Affiliations:** 1 Department of Zoology, University of Córdoba, Campus of Rabanales, 14071 Córdoba, Spain; ba1flsej@uco.es; 2 Department of Environment and Agronomy, National Research Council—Institute for Agricultural and Food Research and Technology, CSIC-INIA, Crta. Coruña Km. 7.5, 28040 Madrid, Spain; 3 International Doctoral School, Doctorate in Science (Environmental Chemistry Line), National University of Distance Education, C/Bravo Murillo 38, 28015 Madrid, Spain; 4 Department of Analytical Chemistry, Physical Chemistry and Chemical Engineering, Faculty of Sciences, Alcala University, University Campus, Ctra. Madrid-Barcelona Km 33.600 E-28871, 28801 Alcalá de Henares, Spain; anotioferalba@gmail.com; 5 Department of Desertification and Geoecology, Arid Zones Experimental Station, EEZA-CSIC, Crta. de Sacramento s/n. La Cañada de San Urbano, 04120 Almería, Spain; hernando.dolores@eeza.csic.es

**Keywords:** beeswax, residues, decontamination, honey combs, *Apis mellifera*

## Abstract

**Simple Summary:**

Honey bees are important for ecosystems and crops, but their populations are declining due to threats such as residues from veterinary and phytosanitary treatments. These residues accumulate in honeycomb wax, posing a risk to bees. Moreover, the constant recycling of old wax worsens the issue as new residues accumulate. To address this, a study explored using methanol extraction to decontaminate the wax. Now, we have assessed the acceptance of decontaminated wax by bees for building new honeycombs. The results showed that bees accepted it, albeit with a slight delay compared to commercial wax. Therefore, using decontaminated wax may be a viable solution to mitigate hazards from high residue levels.

**Abstract:**

Honey bees face serious threats. These include the presence of the *Varroa destructor* mite in hives, which requires the use of acaricides to control. The constant recycling of old wax exacerbates the problem, and results in the accumulation of residues in the beeswax, which is a problem for the viability of the colony. The same happens with the accumulation of phytosanitary residues. In a previous study, we implemented an efficient wax decontamination method using a batch methanol extraction method. The present study evaluates the acceptance of the decontaminated wax by the bees for comb building, brood, honey and pollen containment. The results show a slight delay in the start of comb building and small changes were observed in the pharmacopoeia of the decontaminated wax compared to the original commercial wax. The slight delay in the acceptance of the decontaminated wax could be due to the loss of some components, such as honey residues, which usually appear in the wax. The addition of bee-attractive substances to the manufacturing process could help to mitigate the delay. The results suggest that the use of decontaminated wax is a good alternative to reduce the concentration of residues in hives.

## 1. Introduction

Our evolution as a human society owes a significant debt to honey bees. Since ancient times, we have benefited from products such as honey, pollen, wax, and royal jelly. Most importantly, we have relied on their pollinating activities for the conservation of ecosystems and the pollination of multiple crops [1]. Among bees, the most studied and well known is the western honey bee (*Apis mellifera* L.). This species, which has accompanied us in our own evolution, is currently facing serious threats such as climate change, habitat loss, harmful parasites such as the *Varroa destructor*, and residues from agricultural pesticides and veterinary treatments, some of which are used for controlling diseases in bees themselves [2].

The bee colony generates its own configuration, consisting of a structural component, which are the combs, naturally built from wax scales secreted by worker bees, originating from glands located on the ventral side of the abdomen. Afterwards, the wax scales are manipulated and used to construct hexagonal cells, which will serve to contain the brood and reserves of honey and pollen. The other part of the colony includes the animals and reserves of honey (reserves of carbohydrates) and pollen, in the form of bee bread (a source of proteins, vitamins, and other bioactive components). Naturally, the combs are built as parallel structures, resembling book pages, with cells on both surfaces and separated from each other by a relatively constant space of 1.8 cm. The combs are attached by bees to the upper part of the hive, and to a lesser extent to the sides, leaving connecting bridges between the combs to give more consistency to the structure. On these combs, we find adult bees distributed into three castes: the queen, workers, and drones [3,4]. In the cells, there will be brood, pollen reserves, and honey reserves, arranged in a particular way, in the form of concentric ellipsoid spheres, reserving the central part of the sphere for brood, surrounded by pollen, and more externally, honey. In the artificial handling of bee hives, the same structure previously described is maintained, but each honey comb is contained in a rigid frame, which allows for its manipulation and removal from the hives during management tasks, such as inspections or honey harvesting. In beekeeping, in order to support each honey comb in the frame, and direct the bees in the construction of the honey comb, pre-manufactured wax sheets are fixed with wires, with the printed pattern of the bottom of the cells. These sheets are known as comb foundations. On them, the bees will build new honey combs, even using part of that wax for the start of construction, and later adding more wax produced by them [3].

Comb foundations are industrially produced from recycled natural wax obtained from older honey combs. The wax is melted by heat and filtered to obtain clean wax. This method allows for the repeated reuse of wax, which prolongs its use and saves much of the work of the bees in building honey combs. Unfortunately, this process is not as simple as it seems. Beeswax is primarily composed of compounds such as alkanes, free fatty acids, monoesters, diesters, and hydroxy-monoesters [5]. It is a matrix that accumulates lipophilic substances [6,7,8], including residues from agricultural and livestock treatments such as coumaphos, pyrethroids, and other common substances used to control bee diseases, as well as those from external sources [9,10,11,12,13,14,15]. These substances cannot be eliminated in the usual treatment of wax recycling, and thus they accumulate and increase with each new cycle of wax use, reaching concentrations that pose a threat to bees. Additionally, the manipulation of wax by bees causes these substances to recirculate within the colony. In some instances, residues are transferred to the contents of cells, such as brood or bee bread, which are then consumed by bees and can cause serious problems [15,16,17,18,19,20,21,22]. The previous circumstances have generated a significant problem, which makes it urgent to reverse the process of accumulation of residue in beeswax, for the sake of the survival of bees and for ourselves, as these residues can somehow reach human consumption. Various methods of decontaminating beeswax have been proposed. In laboratory trials, Calatayud-Vernich et al. [23] removed an average of 95% of the contaminants recorded in the wax with several washes using N, N-Dimethylformamide. The method has only undergone testing in a laboratory setting, using hexane-diluted solutions of beeswax at a concentration of 6 % *w*/*v* and a volume of 250 mL. While the decontamination outcomes are promising, it still has a long way to go before it can be considered suitable for use in an industrial-scale process. Other techniques have used filters with different solid adsorbent substances such as diatomaceous earth and activated carbon, with uneven results depending on the type of residues being treated [24,25]. Despite the fact that the amount of residues is decreased, the treatment still results in the wax becoming darker and the loss of volatile components. This may potentially impact the acceptance of bees and hinder the usual operations of the colony [25]. Another lab-scale process removes coumaphos and tau-fluvalinate acaricides from beeswax with a removal efficiency of around 98%. However, the effectiveness of the process on a larger scale is yet to be proven. Additionally, the energy balance of the post-extraction operations, which include cooling, vacuum filtration, and evaporation or vacuum distillation, needs to be evaluated. Ethanol, used in the process, modifies the quality parameters of the beeswax and its cost could hinder its use on a larger scale [26].

A new technique has been developed for decontaminating beeswax, in which the quality of the obtained decontaminated beeswax has similar properties to those of virgin beeswax [27]. The study describes a batch mode procedure, which involves treating the beeswax with methanol at 65 °C followed by an adequation process with water at 70 °C. This procedure was effective in removing pesticide residues from beeswax, with reductions in pesticide residue levels ranging from 87% to 99%. Up to 80% of the methanol used in the extraction was recycled, and the waste generated was easy to manage. The study also determined optimal operating conditions for scaling up the process to an industrial level, using basic tools commonly used in the chemical industry. However, it is important to consider that the removal of pesticides and other chemical substances from beeswax through chemical processes can affect some of its pharmacopoeia properties, which could impact the acceptance of the wax by bees, causing issues in comb construction. Therefore, the aim of this study is to investigate the acceptance by bees of the decontaminated wax obtained by the previously mentioned methanol extraction method.

## 2. Materials and Methods

### 2.1. Sample Collection

The research was carried out during April and May (spring season) of 2020, in an experimental apiary at the University of Córdoba (Córdoba, Spain. 41°44′30.519′′ N, 6°16′41.3322′′ W). The trial was conducted with colonies of the native honey bee (*Apis mellifera iberiensis*, Engel) with young freely-mated queens. The honey bee colonies were housed in Langstroth hives with ten combs. The bee hive contained approximately eight combs with adult bees, bee brood, and food reserves. For the study, two types of wax were used: (i) commercial wax purchased from an apiary store. This wax acted as a control and (ii) the same wax that underwent the decontamination process described by Luna et al. [27].

### 2.2. Extraction and Adequation Processes

The beeswax decontamination process involves two main steps: the extraction process and the adequation process. During the extraction process, beeswax is melted at 65 °C and then treated with methanol to extract contaminants during 30 min, followed by centrifuging and distilling the methanol phase. The resulting product is called “Wet decontaminated beeswax”. In the adequation process, the Wet decontaminated beeswax is dispersed in water at 70 °C during 30 min, decanted to separate the beeswax phase from the water phase, pressed, and dried to obtain “Decontaminated beeswax”. More details of the process can be found in the publication by Luna et al. [27]. The decontamination process was carried out at the facilities of the University of Alcalá de Henares, at the Centre for Applied Chemistry and Biotechnology (CQAB), where the industrial process was implemented with an amount of 75 kg of wax.

### 2.3. Pharmacopoeia Specifications

Both the original commercial wax and the same wax after decontamination were analyzed to determine the changes in quality according to the Royal Spanish Pharmacopoeia [28]. The established physicochemical parameters are as follows: the wax is a non-crystalline yellow solid with a weak honey smell. It must be partially soluble in hot alcohol and ether, and completely soluble in fatty oils. Its melting range (°C) should be between 61 and 66 °C. The viscosity at 100 °C (mPa s) should be <20 and its ash content (%) should be <0.1. The iodine value (g I^2^/100 g wax) should be between 8 and 15, the acid value (mg KOH/g) between 17.0 and 22.0, the saponification value (g KOH/g) between 87 and 102, the ester value (mg/KOH/g) between 70 and 80, and the ester/acid ratio between 3.3 and 4.3.

For the viscosity calculation, approximately 20 g of wax was melted directly in the cylinder of the viscometer (Schott’s Visco Easy Plus), using the low viscosity adapter (LCP). The measurement conditions were set at 100 °C with variable agitation depending on the sample characteristics (less than 60 rpm), until the measurement was stable (5–10 min). Total ashes were measured on 1g of pulverized product in a porcelain crucible, calcined for 4 h at 500 °C. It is important to distribute the product at the bottom of the crucible and heat it, avoiding the formation of isolated charred masses or overflow. The analysis must be performed in triplicate. The melting range was determined by heating the wax over a water bath, pouring it onto a glass plate, and allowing it to cool to form a semi-solid mass. The metal container was then filled by inserting the wider end into the beeswax and repeating the process until the wax was expelled through the narrower opening. Excess was removed with a spatula, and a thermometer was immediately inserted. It was left to rest at room temperature before determining the drop point. For the acid value (*AV*), 2 g of wax were weighed in a 250 mL round bottom flask coupled to a condenser, and dissolved with 40 mL of xylene with constant stirring and reflux heat until the substance dissolved. 20 mL of ethanol and 0.5 mL of the phenolphthalein solution were added, and it was heated with 0.5 M alcoholic potassium hydroxide until a red color persisted for at least 10 s (*n*1, volume in mL). A blank test was performed (*n*2, volume in mL), which was equal to 0.001 since it was a drop; *m* is the mass of wax. The calculation was performed using the following formula:

$$AV\left(\frac{mg\;KOH}{g}\right)=\frac{28.05\left(n1-n2\right)}{m}$$

For the determination of the saponification value (*SV*), 2 g of wax were weighed in a round-bottom flask coupled to a reflux condenser. It was dissolved in 30 mL of a mixture of equal volumes of ethanol/xylene and stirred constantly while heating until the sample was completely dissolved. 25.0 mL of 0.5 M alcoholic potassium hydroxide was added, and it was refluxed for 3 h. The hot solution was then titrated with 0.5 M hydrochloric acid, using 1 mL of phenolphthalein solution as an indicator (*n*1, volume). The solution was reheated to boiling several times during titration. A blank test (*n*2, volume) was also performed:

$$SV\left(\frac{mg\;KOH}{g}\right)=\frac{28.05\left(n2-n1\right)}{m}$$

The ester value is determined as the difference between the saponification index and the acidity index, and is represented as the concentration of potassium hydroxide consumed in the saponification of esters. On the other hand, the ester/acid ratio can be determined by dividing both indices. For the determination of the iodine value (IV), 1 g of the sample was weighed accurately to 0.1 mg using a glass weighing boat. The sample was then transferred to a 500 mL flask and the fat was dissolved by adding 20 mL of the solvent (acetic acid + hexane). Next, exactly 25 mL of Wijs reagent was added to the flask, and the contents were capped, shaken, and placed in the dark. A blank test was prepared using the solvent and reagent, but without the sample (*n*2, volume), in the same manner. The flasks were kept in the dark for 1 h. Afterwards the appropriate time had elapsed, 20 mL of 100 g/L potassium iodide solution and 150 mL of distilled water were added to each flask. The yellow color produced by iodine was titrated with sodium thiosulfate (Na_2_S_2_O_3_) solution until it had almost disappeared. A few drops of starch paste were added, and the titration was continued until the blue color disappeared after very intense agitation (*n*1, volume). The formula is as follows:

$$IV\left(\frac{g\;I^{2}}{100g}\right)=\frac{1269\left(n2-n1\right)}{m}$$

### 2.4. Management of Honey Bee Colonies

Finally, both waxes (commercial wax and decontaminated wax) were laminated and stamped in beekeeping facilities collaborating with the University of Córdoba. After, two trials were carried out:

Trial 1.

**Figure 1:**
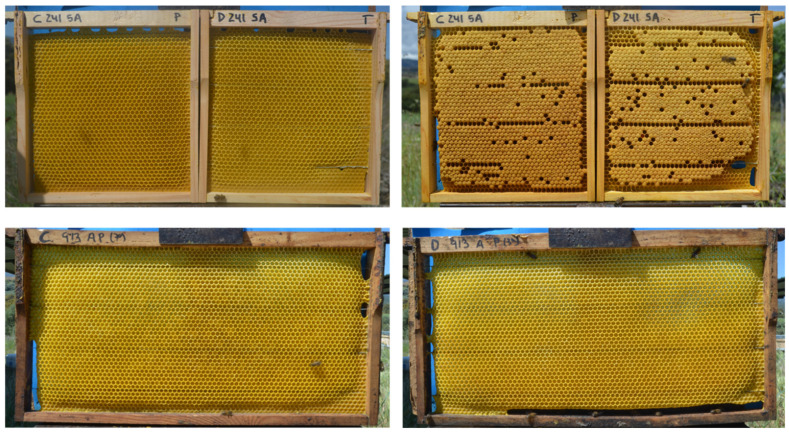
Wax distribution in the tests. In test 1, both types of waxes are next to each other as half-parts of the same frame, identified with “C” the commercial wax, and with “D” the decontaminated wax. On the top left is the beginning of the construction and on the right is the same comb with an advanced stage of brood. In trial 2, each type of wax occupied a complete frame. Below left commercial wax and on the right is decontaminated wax.

In this trial, the comb foundations of both types of wax were fixed in the same frame, using two half-frames of Langstroth hive, which were joined together. In this way, the commercial wax and decontaminated wax sheets were placed adjacently (view Figure 1). Four hives were used for this trial. Two complete frames were placed in each hive, each with the two types of wax. The frames were placed in positions 5 and 9. Each frame and each type of wax was duly identified on the top of the frame with the identification of the hive, the position, the face of the frame, and the type of wax used. The hives were then inspected at 24 h, 48 h, 96 h, 168 h, 216 h, 264 h, 336 h, 384 h, and 432 h. These hours are used because at 24/48 h, we can see an initial rapid response from the bees. After that, the checking is extended in order to cause the least discomfort and risk to the bee hive. At each inspection, the frames were removed, the bees were brushed, and a picture of both sides of each comb without bees was taken (Nikon reflex camera D5100; 18/55VR lens, Nikon Europe B.V.). The photographs were later processed with image analysis software (Image J^®^ 1.52k). The start of comb construction, the start of egg laying, the start of brood capping, brood area, honey area and pollen area were recorded in each evaluation. Brood, honey and pollen area were then multiplied by 4 to obtain the number of cells dedicated to each (One cm^2^ ≈ 4 cells. Preliminary studies).

Trial 2.

In trial 1 both types of wax were next to each other, in the same frame, and building combs on one type of wax could induce the bees to build on the other type of wax. In this second trial, comb foundations from each type of wax were placed in a separate complete frame (Figure 1), and both frames were introduced into the hives separated by other combs, at positions 3 and 7. In this trial, five other hives were used, and in each hive, both types of wax were introduced. The position of the two waxes was alternated in the different hives. The rest of the tasks were the same as in trial 1, and the same variables were measured.

### 2.5. Statistical Analysis

Data were statistically processed using SPSS (Statistical Package for the Social Sciences) Statistics software for Windows, IBM Corp, 2016. Version 24.0. IBM Corp, Armonk, NY, USA. The factors considered were the type of wax used (commercial and decontaminated), the trial (trial 1 and trial 2), the position of each frame within the hive (positions 5 and 9 in trial 1 and 3 and 7 in trial 2), each side of each comb (side A and side B) and the hive (1A to 1D in trial 1 and 2A to 2E in trial 2). The number of brood cells, pollen, and honey recorded in each evaluation were considered as dependent variables.

## 3. Results and Discussion

The data did not fit a normal distribution for any of the variables studied: area built with brood, honey or pollen, so non-parametric statistics were used. The tests are specified together with the results.

Trial 1.

**Figure 2:**
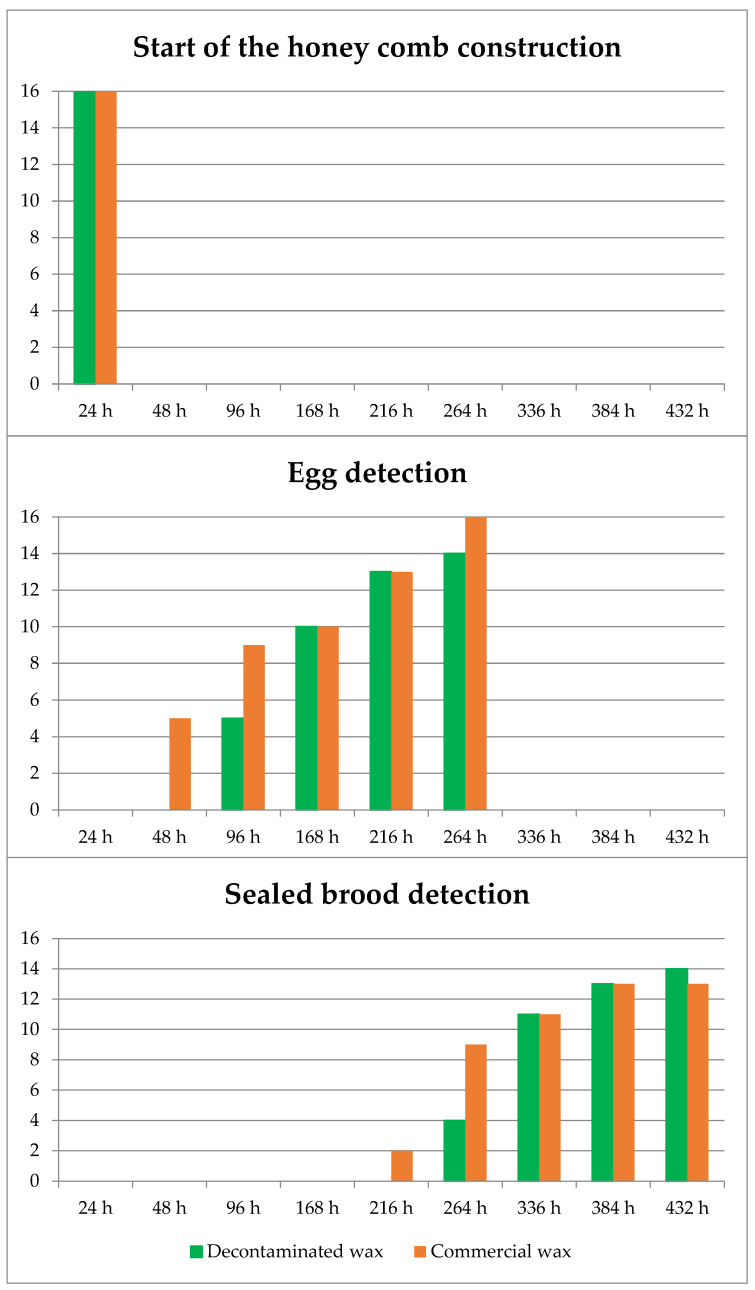
Trial 1. Number of surfaces of half combs where bee activity was detected over time: start of comb construction, start of egg laying and start of brood capping.

In trial 1, a total of 16 half-frame surfaces were evaluated for each wax type (4 bee hives × 2 half combs/bee hive × 2 sides/half comb). Twenty-four hours after the introduction of the comb foundations into the hives, comb construction had started on all combs in both wax types. However, the construction was significantly larger in the commercial wax (mean ± s.d.) (288.2 ± 91.0) versus the decontaminated wax (244.9 ± 108.9) (Mann–Whitney U test, *p* ≤ 0.05). After, at the 48 h inspection, no significant differences were found between the surfaces built on both types of waxes, with 303.6 ± 37.8 cm^2^ and 268.9 ± 68.5 cm^2^ for the commercial and decontaminated waxes, respectively. Since it is necessary for the new comb cells to be in an advanced stage of construction for the queens to lay eggs in them, the delay in the start of construction in the decontaminated wax probably affected the start of egg laying in the cells, so that after 48 h, laying was already detected in five half-surfaces of commercial wax compared to none in the decontaminated wax, and after 96 h laying was detected in the combs built on decontaminated wax. At this time, laying was found on 9 surfaces of commercial wax compared to 5 surfaces of decontaminated wax. After 168 h, laying was detected equally on both types of wax. The detection of capped brood occurred after 216 h on two commercial wax surfaces. At 264 h, on nine surfaces of commercial wax and four of decontaminated wax, and from 336 h onwards, capped brood was detected on 11 surfaces of each of the waxes. Finally, at 432 h, capped brood was detected on 14 surfaces of commercial wax and 13 surfaces of decontaminated wax. The results are shown in Figure 2.

**Figure 3:**
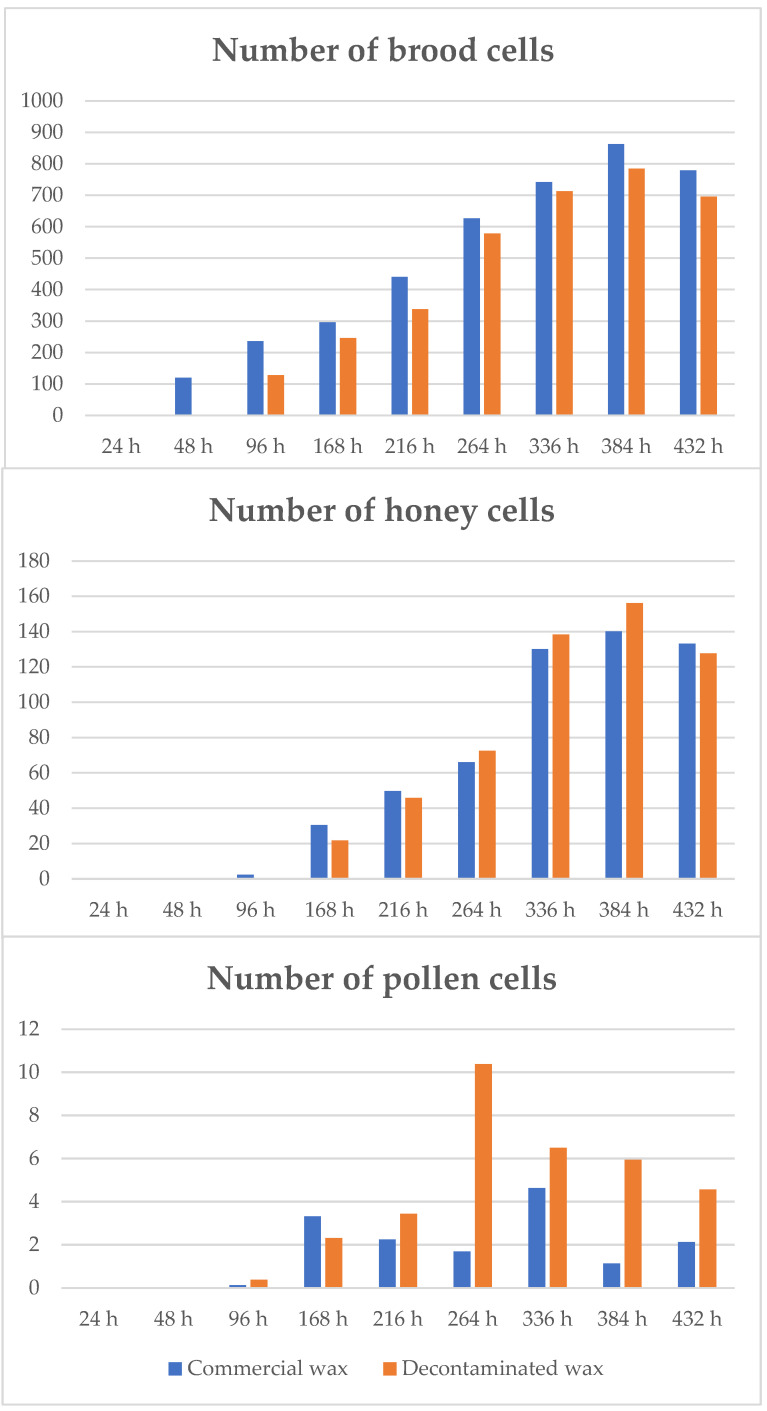
Average number of cells occupied with brood, honey and pollen in combs constructed from comb foundations of commercial wax and decontaminated wax in each of the hive inspections (hours).

On the other hand, the number of cells that the bees spent on brood, honey and pollen was also measured for each inspection. The results are shown in Figure 3 (more details in Table S1). Most cells were occupied by brood, followed by honey and finally pollen. The results showed a mean for all hives and all evaluations of (mean ± s.d.) 512.6 ± 390.5 and 435.3 ± 387.5 brood cells, 69.0 ± 129.0 and 70.4 ± 128.5 cells with honey and 1.9 ± 6.8 and 4.2 ± 11.4 cells with pollen for commercial wax and decontaminated wax combs, respectively.

No significant differences were found between the two types of wax for either the number of brood cells or honey cells. Instead, in the decontaminated wax combs, the bees deposited pollen in significantly more cells than in the commercial wax combs (Mann–Whitney U test, *p* ≤ 0.05), although the amount was really small. The above results indicate that, although the start of comb construction on decontaminated wax was slightly delayed compared to commercial wax, and this may have affected the start of egg laying, the final acceptance was good and did not significantly affect the bees’ use of the cells.

Other factors that could influence comb construction and use, such as the position of the frames or the side of the comb, were also considered. As expected, significantly more brood was detected in frame position 5 than in frame position 9 throughout most of the trial. In contrast, significantly more brood was only detected in the commercial wax versus the decontaminated wax in the 48 h assessment in the frames at position 5. Additionally, no significant differences in brood quantity were detected in the frames at position 9 in any assessment. Furthermore, no significant differences were detected between the number of cells used by bees for brood, honey or pollen between the two comb surfaces (sides A and B) (Mann–Whitney U test, *p* ≤ 0.05).

Overall, the results of this trial showed that both commercial and decontaminated waxes were well accepted by the bees, although a delay in the start of comb construction on decontaminated wax could be observed, and therefore, there was a delay in the start of laying.

Trial 2.

Since in trial 1 the comb foundations of commercial wax and decontaminated wax were placed next to each other in each frame and the activity of the bees on one wax could influence the other half, it was decided to test with each type of wax in different frames and separated by other combs. In this case, the whole of each comb corresponded to one type of wax, and two complete surfaces (sides A and B) were obtained from each comb. In total, there were 10 surfaces for each wax type (5 hives × 1 frame/hive × 2 surfaces/frame). The results showed that after 24 h comb construction had started on all sheets, irrespective of the type of wax used on the foundations, the position of the frame in the hive, or the side of each frame. In contrast to trial 1, no significant differences were detected in the surface area under construction on comb foundations of both wax types, although the cells could be in different degrees of construction (Mann–Whitney U test, *p* > 0.05), with a mean (mean ± s.d.) surface area of 509.0 ± 28.5 cm^2^ and 511.9 ± 181.1 cm^2^ for the commercial and decontaminated waxes, respectively. Although the constructed surfaces were very similar for both types of waxes, in the case of the decontaminated wax the variability (measured as standard deviation) was much higher, indicating greater differences in the speed of construction of these combs in the different hives. On the other hand, the placement of the foundations in different positions within the hives should also have had an effect, since bees tend to be more active in building combs in the central areas of the swarm versus more peripheral areas. The fact that the positions of each type of wax in the different hives were changed may also have influenced the fact that it detected fewer differences between the two types of waxes.

During the 48 h inspection, the use of the constructed cells was detected. At this time, egg laying had begun on 4 surfaces of commercial wax and 1 of decontaminated wax. At 96 h, eggs had been laid on 6 commercial and 3 decontaminated wax surfaces. At 168 h, eggs were laying on all commercial wax surfaces, while it was necessary to wait until 264 h before they also appeared on all decontaminated wax surfaces. Similarly, to what was found in trial 1, the decontaminated wax was accepted by the bees, although with a certain delay compared to the original commercial wax.

When cell usage was considered, it was found that, as in trial 1, the highest number of cells was dedicated to brood, with a mean occupancy for all evaluations (mean number of cells ± s.d.) of 1376.0 ± 690.9 and 1105.4 ± 803.0 brood cells, 155.8 ± 192.8 and 104.5 ± 146.2 cells with honey and 4.7 ± 12.0 and 10.5 ±17.8 cells with pollen for the commercial wax and decontaminated wax combs, respectively. Although a higher number of brood and honey cells is observed in commercial versus decontaminated wax combs, no significant differences were detected (Mann–Whitney U test, *p* > 0.05). On the other hand, a significantly higher number of cells was dedicated to pollen storage in the decontaminated versus commercial wax combs (Mann–Whitney U test, *p* ≤ 0.05), as was the case in trial 1, and in this case also the number of cells was really low, as it should occur in a period of intense colony population growth, when food resources, especially pollen as a source of protein for brood feeding, are rapidly consumed. On the other hand, the high variability (presented as standard deviation) shown by the data, especially for pollen and honey cells, should be due to the dynamics of the colonies, changing more frequently the use of the cells according to the needs, which occurs less frequently in brood cells, which have to wait until the birth of the new bees to change the use that can be given to them.

When the use of the cells by the bees was compared, no significant differences were detected between the two types of waxes for any of the contents, bee brood, honey, or pollen, in any of the evaluations (Mann–Whitney U test, *p* > 0.05).

The results of the second trial confirmed those found in trial 1, indicating a good acceptance of the decontaminated wax, although with some delay compared to the original commercial wax.

The explanation for the delay in starting work on decontaminated wax should be sought in possible alterations of the wax attraction, due to the residue extraction process with methanol. In the pharmacopoeia study of the wax, it is noted that minor alterations were found in the decontaminated wax compared to the initial commercial wax (Table 1). However, it is likely that the reduced attractiveness of the decontaminated wax to bees is mainly due to the loss of other characteristics, such as odor, or even flavor. Since in the decontamination process the wax goes through methanol and water washes, different substances, such as some volatiles compounds or traces of honey that give a characteristic odor, are lost, making recognition by bees more difficult. Following this study, if it were really necessary to accelerate the process of acceptance of the decontaminated wax by the bees, different solutions could be proposed. As an example, an easy solution could be to add some flavoring or simply some honey to the wax melting process at the time of manufacturing the comb foundations.

## 4. Conclusions

The decontamination of beeswax is a crucial step towards reducing the hazards faced by honey bees. Luna et al. [27] have demonstrated the effectiveness of their methanol extraction decontamination process in reducing wax residues. The study found that the decontaminated wax was well accepted by bees, with only a slight delay in the start of comb construction compared to commercial wax. This delay was deemed manageable and compensated by the benefits of reduced residue levels.

The findings suggest that beekeepers could benefit from using decontaminated wax for their bees, which could reduce the risks associated with pesticides and other contaminants found in beeswax. The addition of bee-attractive substances to the manufacturing process of comb foundations could also help mitigate the slight delay in comb construction, making it more appealing to beekeepers.

In conclusion, the decontamination of beeswax is an important step towards ensuring the health and well-being of honey bees. The use of decontaminated wax could potentially reduce the negative impact of contaminants on bee colonies, which could result in increased hive productivity and overall bee population.

## Figures and Tables

**Table 1:** Pharmacopoeia parameters of the original commercial wax and the same wax after decontamination with methanol extraction.

	Melting Range (°C)	Viscosity at 100 °C (mPa s)	Ash Content (%)	Iodine Value (g I_2_/100g Wax)	Acid Value (mg KOH/g)	Saponification Value (mg KOH/g)	Ester Value (mg KOH/g)	Ester/Acid Ratio
**Commercial wax**	61–66	9.88	0.04	13.69	20.5	87.32	66.86	3.26
**Decontaminated wax**	61–66	10.01	0.04	13.46	17.24	84.48	66.91	3.91

## Data Availability

No new data were created or analyzed in this study. Data sharing is not applicable to this article.

## Supplementary Materials

The following supporting information can be downloaded at: https://www.mdpi.com/article/10.3390/insects14070593/s1, Table S1: Number of cells constructed on comb foundations of commercial wax and decontaminated wax, and occupied by brood, honey or pollen.
