# Supplementary material for: Acceptance by Honey Bees of Wax Decontaminated through an Extraction Process with Methanol
Source: Insects. 2023 Jun 30;14(7):593. doi: 10.3390/insects14070593 (PMC10380593; doi:10.3390/insects14070593)
Supplement: Supplementary file 1 [file insects-14-00593-s001.zip › insects-2466696-supplementary.pdf]

Table S1. Number of cells constructed on comb foundations of commercial wax and decontaminated wax, and occupied by brood, honey or pollen. In trial 1, half contiguous frames of both wax types were used. In trial 2, full frames were used for each wax type. Data are given as the number of observations (N) and the mean (mean ± s.d.) of the number of cells occupied by brood, honey and pollen in each assessment (hours), as well as the total mean of the assessments.

	**USE OF THE CELLS**	**WAX**	**48h**		**96 h**		**168 h**		**216 h**		**264 h**		**336 h**		**384 h**		**432 h**		**Overall average**	
			N	Mean ± s.d.	N	Mean ± s.d.	N	Mean ± s.d.	N	Mean ± s.d.	N	Mean ± s.d.	N	Mean ± s.d.	N	Mean ± s.d.	N	Mean ± s.d.	N	Mean ± s.d.
TRIAL 1	NUMBER OF BROOD CELLS	Commercial wax	16	119.8 ± 186.5	16	235.8 ± 268.0	16	295.5 ± 265.1	16	440.1 ± 273	16	626.4 ± 347.2	16	741.6 ± 393.2	16	862.8 ± 290.6	16	779.1 ± 318.5	128	512.6 ± 390.5
		Decontaminated wax	16	0.0 ± 0.0	16	128.2 ± 216.9	16	246.1 ± 217.8	16	337.7 ± 235.7	16	578.6 ± 325.2	16	712.3 ± 364.8	16	784.4 ± 355.3	16	695.1 ± 315.5	128	435.3 ± 387.5
		Total	32	59.9 ± 143.3	32	182.0 ± 246.0	32	270.8 ± 240	32	388.9 ± 256.2	32	602.5 ± 331.8	32	727 ± 373.4	32	823.6 ± 321.8	32	737.1 ± 314.7	256	474 ± 390.2
	NUMBER OF HONEY CELLS	Commercial wax	16	0.1 ± 0.3	16	2.3 ± 6.3	16	30.4 ± 71.2	16	49.8 ± 95.3	16	66 ± 91.3	16	130.1 ± 184.8	16	140.2 ± 158.5	16	133.1 ± 182.7	128	69 ± 129
		Decontaminated wax	16	0.0 ± 0.0	16	0.6 ± 1.6	16	21.7 ± 44.1	16	45.9 ± 68.9	16	72.5 ± 121.7	16	138.4 ± 188.1	16	156.1 ± 187.1	16	127.7 ± 134.1	128	70.4 ± 128.5
		Total	32	0.1 ± 0.2	32	1.4 ± 4.6	32	26.1 ± 58.4	32	47.8 ± 81.8	32	69.3 ± 105.9	32	134.3 ± 183.5	32	148.2 ± 170.8	32	130.4 ± 157.7	256	69.7 ± 128.5
	NUMBER OF POLLEN CELLS	Commercial wax	16	0.0 ± 0.0	16	0.1 ± 0.3	16	3.3 ± 7.3	16	2.3 ± 4.9	16	1.7 ± 5.2	16	4.6 ± 13.8	16	1.1 ± 3.7	16	2.1 ± 7.7	128	1.9 ± 6.8
		Decontaminated wax	16	0.0 ± 0.0	16	0.4 ± 0.8	16	2.3 ± 4.7	16	3.4 ± 5.7	16	10.4 ± 23	16	6.5 ± 12.9	16	5.9 ± 13.3	16	4.6 ± 9.2	128	4.2 ± 11.4
		Total	32	0.0 ± 0.0	32	0.3 ± 0.6	32	2.8 ± 6.0	32	2.8 ± 5.3	32	6 ± 17	32	5.6 ± 13.2	32	3.5 ± 9.9	32	3.3 ± 8.4	256	3 ± 9.4
TRIAL 2	NUMBER OF BROOD CELLS	Commercial wax	10	322.7 ± 427.6	10	530.2 ± 463.3	10	1176.1 ± 106	10	1420.3 ± 248.5	10	1528.1 ± 278.7	10	1978.7 ± 281.2	10	1964.2 ± 232.4	10	2088 ± 239	80	1376 ± 690.9
		Decontaminated wax	10	0.0 ± 0.0	10	151.7 ± 322.4	10	662.3 ± 545.9	10	1021.7 ± 635.2	10	1367.5 ± 259	10	1781.4 ± 159.2	10	1872 ± 186.1	10	1986.6 ± 181.8	80	1105.4 ± 803
		Total	20	161.4 ± 337.7	20	341.0 ± 434.3	20	919.2 ± 464.7	20	1221.0 ± 512	20	1447.8 ± 274.5	20	1880.1 ± 244.3	20	1918.1 ± 210.3	20	2037.3 ± 213.1	160	1240.7 ± 758.9
	NUMBER OF HONEY CELLS	Commercial wax	10	44.7 ± 75.1	10	267.6 ± 371.9	10	237.8 ± 246.1	10	192.9 ± 154.3	10	144.6 ± 118.9	10	143.6 ± 134.5	10	114.1 ± 123.8	10	101.3 ± 91.1	80	155.8 ± 192.8
		Decontaminated wax	10	17.6 ± 25.2	10	97.9 ± 103.6	10	185.1 ± 234.2	10	154.1 ± 201.5	10	96.6 ± 123.5	10	85.5 ± 115.7	10	90.6 ± 148.8	10	108.5 ± 104.1	80	104.5 ± 146.2
		Total	20	31.2 ± 56.3	20	182.8 ± 279.6	20	211.5 ± 235.4	20	173.5 ± 175.8	20	120.6 ± 120.5	20	114.6 ± 125.7	20	102.4 ± 133.8	20	104.9 ± 95.3	160	130.2 ± 172.5
	NUMBER OF POLLEN CELLS	Commercial wax	10	0.1 ± 0.3	10	10.0 ± 16.8	10	14.4 ± 24.6	10	5.7 ± 11	10	1.7 ± 3.2	10	0.9 ± 1.7	10	0.6 ± 1.1	10	4.4 ± 5.9	80	4.7 ± 12
		Decontaminated wax	10	0.3 ± 0.7	10	14.0 ± 19.2	10	18.2 ± 20	10	21.7 ± 32.9	10	14.2 ± 18.8	10	4.8 ± 8.3	10	4.2 ± 6.7	10	6.4 ± 4.5	80	10.5 ± 17.8

Total	20	0.2 ± 0.5	20	12.0 ± 17.7	20	16.3 ± 21.9	20	13.7 ± 25.2	20	8 ± 14.6	20	2.9 ± 6.2	20	2.4 ± 5	20	5.4 ± 5.2	160	7.6 ± 15.4
